# Utility of the ADAS-Cog as a Cognitive Screening Tool in Older Adults with Epilepsy: A Multicenter Cohort Study

**DOI:** 10.64898/2026.05.27.26354210

**Published:** 2026-05-28

**Authors:** Jessica Kania, Ifrah Zawar, Anny Reyes, Victoria Williams, Rani Sarkis, Vineet Punia, McKenna Williams, Lisa Ferguson, Kayela Arrotta, Robyn M Busch, Jana Jones, Bruce P Hermann, Carrie R McDonald

## Abstract

**Objective:**

Cognitive impairment is common among older adults with epilepsy, although efficient screening tools suitable for routine use are lacking. Here we examine, for the first time, the utility of the Alzheimer’s Disease Assessment Scale-Cognitive Subscale (ADAS-Cog) as a screening tool to identify cognitive impairment in older adults with epilepsy.

**Methods:**

Participants included 83 adults (ages ≥ 55) with epilepsy from the Brain, Aging, and Cognition in Epilepsy (BrACE) study and 83 age-, sex-, and education-matched cognitively healthy controls from the Alzheimer’s Disease Neuroimaging Initiative (ADNI-3). All completed the ADAS-Cog and a comprehensive neuropsychological battery to identify cognitive phenotypes (intact vs impaired). Performance on individual ADAS-Cog items and the total score was assessed, and diagnostic efficiency statistics were determined.

**Results:**

Epilepsy participants (mean age=66.4 years) performed significantly worse across the ADAS-Cog total score and 8 of the 13 individual test items compared to controls. The largest effect sizes were observed on verbal learning and memory tasks, particularly word recall ( *d* =0.87) and delayed word recall ( *d* =1.06). An ADAS-Cog total score of ≥15 yielded optimal diagnostic efficiency (67.5% accuracy, 68.8% sensitivity, 66.7% specificity) for identifying cognitive impairment.

**Significance:**

The ADAS-Cog is sensitive to detecting cognitive impairment in older adults with epilepsy and may represent a scalable screening option in this population. Additional comparative studies in older epilepsy populations are needed to determine the sensitivity of this measure to longitudinal change, cross-cultural applicability, and availability across languages.

**Plain language summary:**

Cognitive decline is common among older adults with epilepsy, although sufficient evidence supporting the use of screening tools to identify cognitive impairment in this population is lacking. The ADAS-Cog may be a useful screening option in epilepsy research and clinical care, although additional studies are needed to compare it with other cognitive screening tests and to confirm its applicability for clinical care and across cultures and healthcare settings.

**Key Points:**

□ ADAS-Cog, a tool widely used in Alzheimer’s disease research, was used to detect cognitive impairment in older adults with epilepsy versus healthy controls.
□ Largest deficits were in verbal learning and delayed recall, aligning with epilepsy-related memory vulnerability.
□ ADAS-Cog was compared with the gold standard, the International Classification of Cognitive Disorders in Epilepsy (IC-CoDE). An ADAS-Cog-13 cutoff ≥15 showed optimal accuracy for IC-CoDE–defined cognitive impairment.
□ ADAS-Cog performance reliably distinguished IC-CoDE-impaired from intact participants within the epilepsy cohort.
□ These findings support ADAS-Cog as a cognitive screening tool in aging epilepsy populations to identify neurodegenerative vulnerability.

## Full Text Availability

The license terms selected by the author(s) for this preprint version do not permit archiving in PMC. The full text is available from the preprint server.

